# A Comprehensive Review of Lithium-Ion Capacitor Technology: Theory, Development, Modeling, Thermal Management Systems, and Applications

**DOI:** 10.3390/molecules27103119

**Published:** 2022-05-12

**Authors:** Danial Karimi, Hamidreza Behi, Joeri Van Mierlo, Maitane Berecibar

**Affiliations:** 1 Research Group MOBI—Mobility, Logistics, and Automotive Technology Research Centre, Vrije Universiteit Brussel, Pleinlaan 2, 1050 Brussels, Belgium; hamidreza.behi@vub.be (H.B.); joeri.van.mierlo@vub.be (J.V.M.); maitane.berecibar@vub.be (M.B.); 2 Flanders Make, 3001 Heverlee, Belgium

**Keywords:** lithium-ion capacitor (LiC), lithium-ion battery, electric double-layer capacitor, electro-thermal modeling, lifetime modeling

## Abstract

This review paper aims to provide the background and literature review of a hybrid energy storage system (ESS) called a lithium-ion capacitor (LiC). Since the LiC structure is formed based on the anode of lithium-ion batteries (LiB) and cathode of electric double-layer capacitors (EDLCs), a short overview of LiBs and EDLCs is presented following the motivation of hybrid ESSs. Then, the used materials in LiC technology are elaborated. Later, a discussion regarding the current knowledge and recent development related to electro-thermal and lifetime modeling for the LiCs is given. As the performance and lifetime of LiCs highly depends on the operating temperature, heat transfer modeling and heat generation mechanisms of the LiC technology have been introduced, and the published papers considering the thermal management of LiCs have been listed and discussed. In the last section, the applications of LiCs have been elaborated.

## 1. Introduction

Currently, the reduction of carbon dioxide (CO_2_) emissions to decrease global warming and the greenhouse gas (GHG) effect is a matter of grave concern, in which the negative impacts of oil depletion, GHG, and vehicular emissions are rising annually [1]. In this regard, by increasing the number of vehicles and rising fuel consumption, there would be a question of the tolerability of environmental pollution. The possible solution has been the investigation of zero-emission vehicles to reduce GHG emissions. Towards this direction, conventional internal combustion engines ought to be replaced in the field of mobility. Environmentally friendly rechargeable ESSs could contribute to this direction, as they can adequately supply an electro-motive application such as hybrid, plug-in hybrid, and pure electric vehicles [2].

Usually, ESSs are mostly comprised of LiBs and EDLCs as the primary electrochemical storage systems. LiBs are manufactured in different gravimetric and volumetric characteristics and can meet the increased demands on energy and power as well as lifetime capabilities [3]. High energy densities and low self-discharge are among the advantages of LiBs. Nevertheless, specific LiB chemistries such as NMC (Lithium-Nickel-Manganese-Cobalt-Oxide), widely used in many applications, encounter challenges when dealing with increased charge or discharge current rates [4]. In addition, some deficiencies have been seen in the case of generating high-power output during acceleration [2]. For instance, some LiBs supply at least 200 Wh/kg of specific energy, but at the same time less than 350 W/kg of specific power [5].

On the other hand, EDLCs have high power densities with a long lifetime that can be charged and discharged quickly but suffer from low energy densities. For instance, some EDLCs supply at least 10 kW/kg of specific power, but at the same time, less than 5 Wh/kg of specific energy [6]. Gao et al. [7] used EDLCs along with LiBs. However, the usage of EDLCs often requires a bulky and highly efficient DC–DC converter, which counteracts the cost and energy efficiency of the system [8]. The logic system can be based on the hybridization of LiBs with EDLCs to achieve higher energy and power values within a longer lifetime [9]. Therefore, the anode electrode material of LiBs and cathode electrode material of EDLCs were combined in a single ESS to develop a hybrid ESS combining the high energy of LiBs and high power and long lifetime of EDLCs [10]. In such a context, lithium-ion capacitor (LiC) came into existence. Despite all the advantages such as high specific power and energy, and long lifetime, LiCs generate excessive amounts of heat in high current rates that should be controlled for safety reasons [11]. Therefore, a robust thermal management system (TMS) is vital for all electrical energy storage systems [12]. In this context, special attention has been paid to thermal modeling in this paper.

## 2. Various Types of Energy Storage Systems

### 2.1. Lithium-Ion Batteries (LiBs)

**Figure 1:**
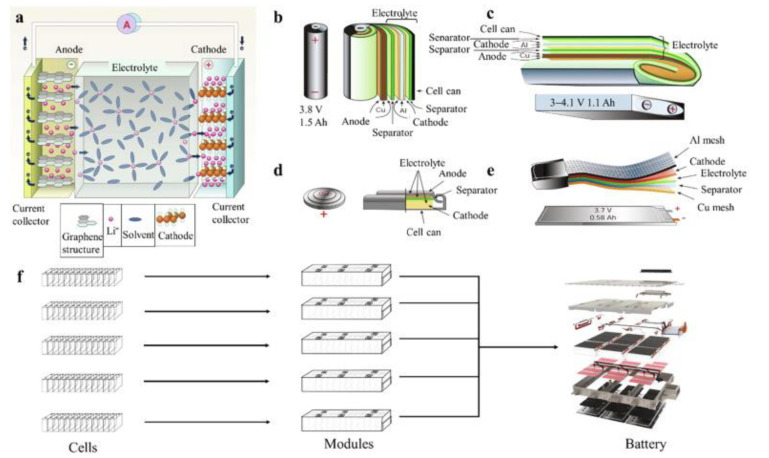
(**a**) A graphical view of the LiB structure for all the different battery types [13]; (**b**) cylindrical cell type; (**c**) prismatic cell type; (**d**) coin cell type; (**e**) pouch cell type; (**f**) the relationship between cells, modules, and battery pack [20].

Lithium-ion batteries (LiBs) consist of four main domains: anode and cathode as the charge carriers, separator to divide electrodes to avoid short-circuits, and electrolyte to carry ions [13]. When LiBs are charged and discharged, electrodes generate heat, which should be controlled to prevent battery malfunction [14]. For example, by damaging the separator, uncontrolled electrochemical reactions would generate significant amounts of excess heat [15]. In such uncontrollable cases, the electrolyte will behave as an additional fuel supply for further heat generation, which will result in thermal runaway. An example of the working principle of LiBs with Lithium-Cobalt-Oxide (LiCoO_2_)/graphite is depicted in Figure 1. During the charging of LiBs, lithium ions de-intercalate from the cathode and diffuse into the electrolyte, which leads to ion movement to the separator to be intercalated into the anode. The electrons move in the same direction as the flow of ions (i.e., from the positive electrode to the negative electrode or vice versa) in the external circuit. Meanwhile, the current flows in the opposite direction to the movement of electrons. During discharging of LiBs, lithium ions move back from the anode to the cathode. The shuttling of lithium ions during charging and discharging generates excessive heat due to the Joule loss and chemical loss [16]. Although the heat generated during battery operation is normal, the battery safety will be compromised if there is no dissipation path to remove the heat loss during charging and discharging processes [17].

### 2.2. Electric Double-Layer Capacitors (EDLCs)

**Figure 2:**
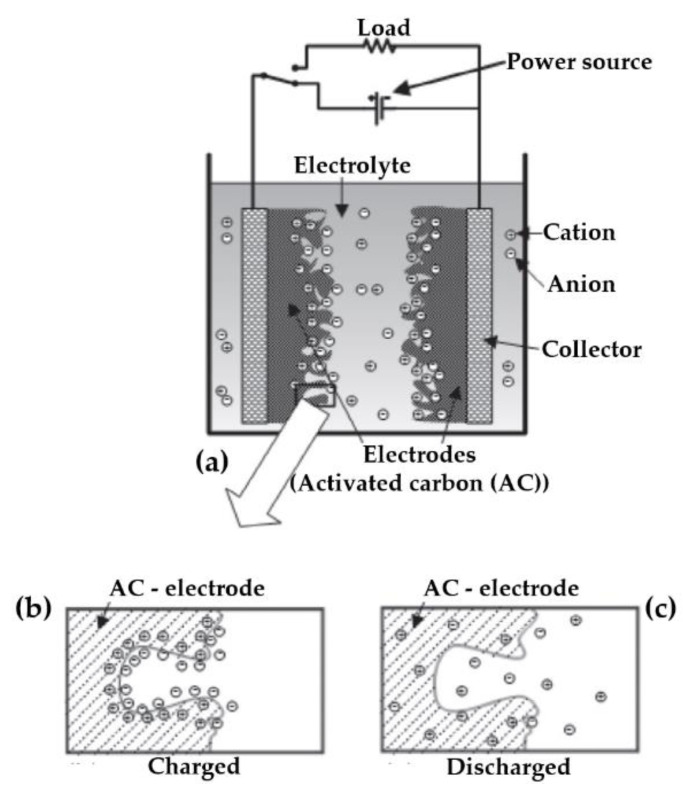
The EDLC formed by a collector, AC electrodes, and an electrolyte: (**a**) concept, (**b**) charging, (**c**) and discharging [18].

Electric double-layer capacitors (EDLC), also known as supercapacitors (SC) or ultracapacitors, store energy in their electric double-layer. In EDLCs, a shorter distance between plates and larger area plates results in higher effective capacitance. The energy storage mechanism of EDLCs is achieved via charge separation at electrode–electrolyte interfaces. The ions are physically absorbed in the surface of electrodes commonly made of activated carbon (AC), carbon nanotubes (CNT), and graphene. Figure 2 shows the basic ides for EDLCs, where AC as two electrodes are placed into an electrolyte [18]. As Figure 2a demonstrates, by applying a power source to AC electrodes, charge layers are formed; positive and negative. These charge layers are oppositely layered to be formed into the electrolyte. AC electrode material is porous activated carbon with high electrical conductivity, high chemical stability, and low cost. In addition, the AC electrode has a large surface area per specific surface area due to the distribution of pores (Figure 2b) for each electrode–electrolyte surface (Figure 2c). Pseudocapacitors can be grouped as supercapacitors with the storage mechanism of fast surface redox reactions occurring between the electrode and the electrolyte [19].

### 2.3. Lithium-Ion Capacitors (LiCs)

**Figure 3:**
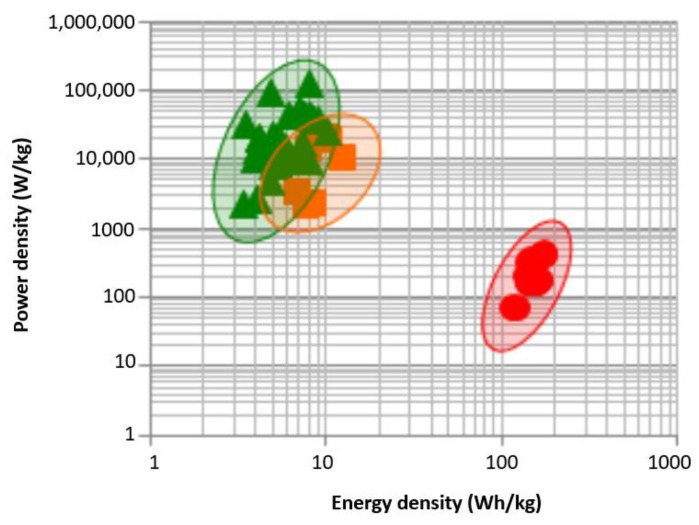
The Ragone plot for different ESSs [24].

The LiC represents an emerged technology that combines the pre-lithiated anode electrode material of LiBs and the cathode electrode material of EDLCs [21]. This electrode combination inherits the high power density and longer lifetime of EDLCs with the high energy density of LiBs [22]. Typically, LiCs show 4 to 5 times more energy than EDLCS due to the utilization of a bulk mechanism to store the energy in one of the electrodes. In addition, LiC’s electrolyte material can achieve higher voltage values compared to standard EDLCs. Therefore, LiCs can compete with EDLCs in terms of energy density. Nevertheless, LiCs have less energy density than LiBs, but can never compete with LiBs in terms of energy density [23]. Although the energy density of LiCs is lower than that of LiBs, the power density of LiCs is much higher than LiBs. Figure 3 exhibits the performance of LiCs in terms of energy and power densities as a Ragone plot [24], in which the specific energy of LiCs is in the range of 25–100 Wh/kg, with a specific power of 1000–10,000 W/kg.

### 2.4. Construction of Lithium-Ion Capacitors

**Figure 4:**
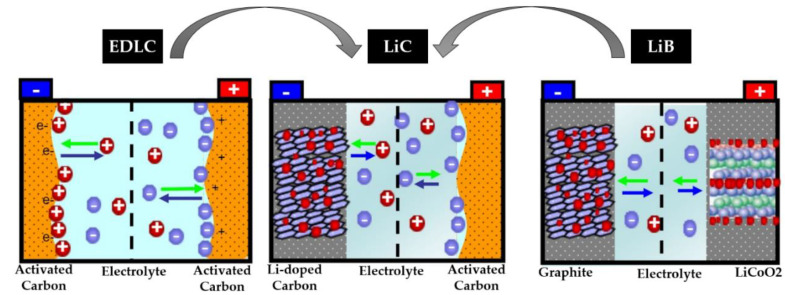
The storage mechanism and chemical structure of LiBs, LiCs, and EDLCs. The blue arrows denote the discharging process while the green arrows denote the charging process [25].

**Figure 5:**
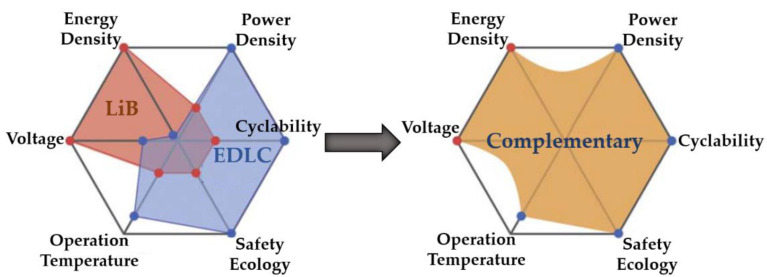
Comparison of LiBs and EDLCs characteristics to form a complementary system [28].

Figure 4 shows the storage mechanism of three different ESSs, including LiBs, LiCs, and EDLCs [25], in which the LiC technology stands between LiBs and EDLCs. As can be observed, although EDLCs have a symmetric construction, the LiC bears an asymmetric configuration as it employs two different types of electrodes. As explained, the charge and discharge processes of EDLCs are based on a non-Faradaic double-layer capacitor [26]. Since LiCs combine two types of electrodes, on one side, anions are adsorbed into/desorbed from the positive electrode’s surface, and on the other side, ions of lithium are intercalated into/de-intercalated from the negative electrode’s body. Faradaic intercalation and non-Faradaic surface reaction are the main reasons for the high power and energy densities of LiCs. The power ($P_{ESS}$ [*W*]) and energy ($E_{ESS}$ [*J = W·s*]) of an ESS can be expressed as [27]:

(1) $$E_{ESS}=\frac{1}{2}CV^{2}$$

(2) $$P_{ESS}=\frac{V^{2}}{4R}$$

where *C* [*F*], *V* [*V*], and *R* [*Ω*] denote the cell’s capacitance, potential, and equivalent series resistance (ESR). Figure 5 demonstrates the characteristics of LiBs and EDLCs over six essential criteria, including energy density, power density, cyclability, safety ecology, operation temperature, and voltage for electrochemical ESSs. A synergistic impact is expected by combining EDLCs and LiBs to form a hybrid ESS such as LiCs.

**Figure 6:**
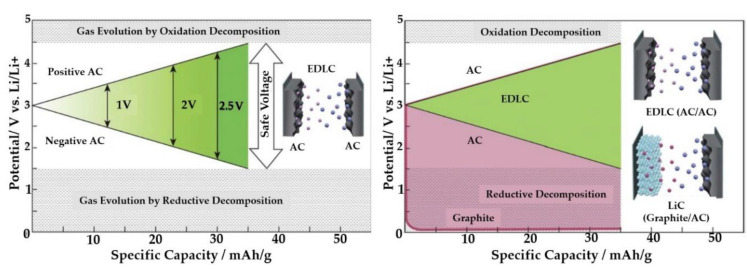
The potential comparison between EDLCs and LiCs [28].

Figure 6 compares the potential window of EDLCs and LiCs to conclude that increasing the voltage will enhance the energy and power densities as they carry a direct proportion. The operation voltage window for two electrode materials of LiCs is different since the electrochemical mechanisms are different. Thus, the voltage window is higher than EDLCs. The negative electrode of the LiC in the figure is graphite, while the positive electrode is AC [28].

In general, EDLCs use AC for both electrodes. The electrolyte is composed of a salt of tetraethyl methyl ammonium tetrafluoroborate (TEMABF_4_) in acetonitrile or propylene carbonate. This commercial EDLC has a maximum voltage of 2.7 V. In contrast, LiCs contain higher voltage ranges up to 4.0 V. Normally, higher voltages for LiC technology can be obtained by setting appropriate materials for positive and negative electrodes. Therefore, the energy density of LiCs is enhanced up to three times compared with EDLCs. In addition, the selection of an electrolyte with a higher potential window leads to improvement of the voltage range. For instance, the aqueous electrolytes of LiCs have voltages between 1.0–1.5 V, while the potential range for ionic liquids and organic electrolytes is up to 4.0 V and 2.5–3.0 V, respectively.

Another essential parameter to enhance the energy density of LiCs is specific capacitance, which can be influenced by the electrode’s extra redox capacitance, the electrolyte and active material’s electrical conductivity, the electrolyte and active material’s pore size distribution, and specific surface area [27]. It is worth mentioning that the specific capacitance calculation directly relates to the effective specific surface area. The effective specific surface area is calculated by cumulative density functional theory. In this theory, the diameter of the pores is larger than the ion in various electrolytes [29].

Thus, the fabrication of nano-porous structures with a highly effective surface area is a practical approach to increasing energy density. In addition, introducing pseudo-capacitive species with heteroatom doping enables to obtain extra redox capacitance, leading to an increase in the LiC’s energy density. As was explained, the operating voltage bears an impact on increasing the energy density, but to increase the power density, ESR is another critical parameter in addition to the operating voltage. The main factors in the ESR include the electrolyte and electrode’s conductivity, the electrode material’s rate of ions, the electrode’s diffusion distance, and the contact resistance between the current collector and electrode material. Increasing the effective specific surface area reduces the ESR resulting in power density enhancement. Moreover, increasing the electrode material’s conductivity and employing an aqueous electrolyte would also increase the power density [30].

## 3. 1D Electrical, Thermal, and Lifetime Modeling

**Figure 7:**
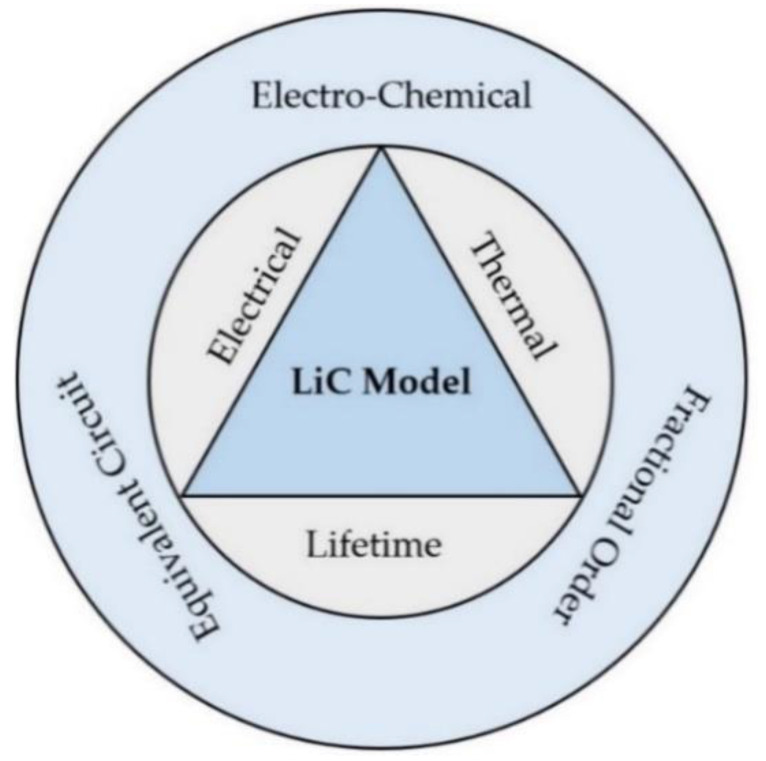
The holistic model of the LiC is applicable for real-time energy management and control purposes [26].

LiC technology is employed at the system level regarding its applications, such as automotive or stationary. Therefore, a holistic modeling approach is needed to extract its electrical and thermal parameters, which will then be used for lifetime modeling [31], safety assessment [32], cell equalization management [33], and thermal management [34]. For example, an accurate electro-thermal model is required for electrical and thermal issues in the developed management system [35]. Moreover, an accurate tool is needed for state of charge (SoC) [36], state of health (SoH) [37], and state of power (SoP) to control the LiC system [38]. The holistic model of the LiC that is applicable for real-time energy management and control purposes is shown in Figure 7 [26].

This section introduces a state-of-the-art review regarding electrical modeling, thermal modeling, and lifetime modeling of LiCs. The LiC model that is developed should be dedicated to a special aim since the goal of modeling is design, parameter identification, state estimation, and control. In this regard, some models, including electrical, thermal, and lifetime models of LiC technology, are presented. The most developed models are electrochemical [39], fractional-order [40], and equivalent circuit models (ECM) [41]. The LiC’s electrical parameters highly depend on temperature, current rate, and SoC. In addition, almost all of the LiC’s characteristics change over the cell’s lifetime [42]. Therefore, a holistic approach should be considered for electrical, thermal, and lifetime modeling under various operating conditions.

### 3.1. Electrical Modeling

The first modeling method is the electrochemical model, which is counted as a powerful tool considering both the physics and chemistry interfaces [43]. Although electrochemical models are accurate, their computational time is high due to modeling of the reaction process by partial differential equations (PDE), which hinders the applicability of these models in energy management and real-time control [44]. Moreover, they can simulate the aging trend of the cell (lifetime estimation) under various working conditions for different ESSs [45,46].

However, this requires a thorough knowledge of the aging mechanism, limiting the applicability of such modeling tools. The most used electrochemical modeling approaches are the lumped parameter model [47] and porous electrode theory [48]. The first approach uses differential-algebraic equations (DAE) to model the electrochemical phenomena inside the cell by considering the chemical products as a uniform spatial distribution. The second approach uses PDE to define the electrochemical processes based on Fick’s law, Ohm’s law, and the Nernst and Butler–Volmer equations [49].

**Figure 8:**
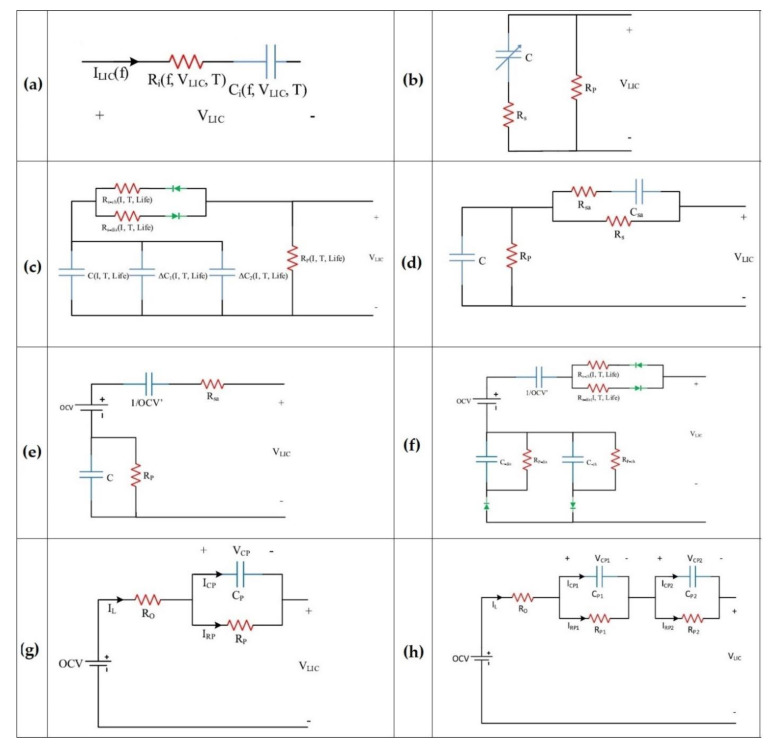
The developed ECMs for the LiC technology [26]: (**a**) R_int_, (**b**) R_int_-R_p_, (**c**) Advanced R_int_-R_p_, (**d**) Hybrid, (**e**) PNGV (Partnership for New Generation of Vehicles), (**f**) Advanced PNGV, (**g**) Thevenin, (**h**) 2-RC.

On the other hand, ECMs are developed under certain conditions from experimental tests, applicable in real-time energy management owing to precision and simplicity [50]. They include RC networks (resistor–capacitor) and utilize ordinary differential equations (ODE) to model the electrical behavior of LiC cells [51]. The main items to enhance the precision of ECMs are circuit configuration and component number [52]. The developed ECMs for the LiC technology are illustrated in Figure 8, in which Figure 8a shows the simplest ECM, including a resistor for overall resistance and a capacitor for the known capacitance impacts, connected in series [24].

Figure 8b depicts a classical ECM for which a parallel resistor is added to the previous ECM to consider the self-discharge phenomenon [53]. Figure 8c illustrates the same topology, in which the overall resistance was split up to the charge/discharge resistances, while three capacitors (C_0_, C_1_, C_2_) are available in the model in parallel connection [54]. C_0_, C_1_, and C_2_ correspond to the initial LiC, capacitor variations at various currents, and capacitor variations at different SoC values. Another critical parameter in the model is the parallel resistance (*R*_*p*_) that is employed for the self-discharge phenomenon:

(3) $$R_{p}=\frac{OCV^{2}\;t_{discharge}}{Wh_{before}-Wh_{after}}$$

$Wh_{before}$ and $Wh_{after}$, and $t_{discharge}$ denote the available energy of the full-charged cell before starting the test, the available energy after the cell is stored at 25 °C for seven days, and discharge time. The main drawback of this ECM is depiction of the cell dynamics only for a short period of several seconds. Figure 8d demonstrates an ECM for power electronics applications that consist of five parameters [55]. One of the model parameters is the capacitance that depends highly on voltage variations to reduce the simulation time. The influence of the internal resistance in series connection with the RC component would be a variation of open-circuit voltage (OCV) at the end of each pulse. The internal capacitance was replaced by a voltage source that is highly dependent on the SoC values to update the model [56]. Figure 8e illustrates the FreedomCAR model that combines the voltage source with the internal capacitor.

Figure 8f illustrates an advanced model of the FreedomCAR for which the current direction is considered. The first-order Thevenin model is one of the most popular ECMs used for various ESSs [57]. This ECM is seen in Figure 8g for LiCs [58].

The variations of capacitance and ESR are evaluated by performing some experimental tests. This model poorly reflects the LiC behavior in diffusion steps and charge transfer, which is the main shortcoming of the first-order Thevenin model. Therefore, the second-order Thevenin model is proposed to cope with this problem to predict the LiC’s transient behavior [59]. This model is shown in Figure 8h. For almost all the mentioned models, the parameters of the models significantly depend on temperature, current rate, and SoC [60]. Moreover, the model’s number of RC branches defines its complexity [61]. If the parameters of ECMs are identified through experiments, the model is called a semi-empirical model [62].

However, other approaches for modeling can be found in the literature [63]. Characterization and parameter identification is the most significant step to develop an ECM [64]. In this context, online and offline methods should be used to identify the electrical and thermal parameters of the cell. Offline methods use lookup tables to store the extracted parameters [65], or the parameters should be fitted using fitting techniques [66]. Among the offline methods, hybrid pulse power characterization (HPPC) [67] and electrochemical impedance spectroscopy (EIS) [68] are the main approaches. As mentioned earlier, the electrical parameters of an ECM highly depend on the SoC values. Therefore, SoC estimation methods should be employed as well to characterize the cell [69]. Coulomb counting method is one of the simplest SoC estimation methods with high accuracy that uses charge and discharge capacity tests to calculate the cell’s capacity [70]. Nevertheless, offline methods are incapable of including the temperature and aging conditions [71]. In contrast, online methods extract the parameters in real-time operation [72]. Among the online methods in the literature, the Kalman Filter (KF) [73], Extended KF (EKF) [74], Adaptive KF (AKF) [75], least square method (LSM) [76], Relevance Vector Machine (RVM) [77], Support Vector Machine (SVM) [78], Neural Network (NN) [79], and Particle Filter (PF) [80] are the most widely used.

**Figure 9:**
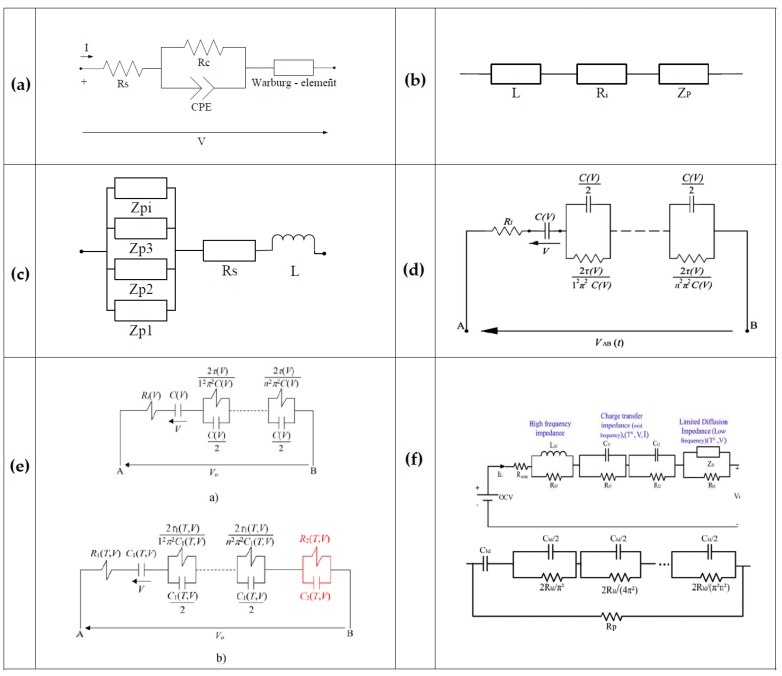
The developed fractional-order models [26]. (**a**) a basic FOM, (**b**) ECM with three components in series, (**c**) the same ECM with four parallel impedance branches, (**d**) developed FOM, in which the impedance is modeled by considering the ambient temperature at various currents (**e**) an enhanced FOM regarding the influence of the cell’s temperature, (**f**) an advanced FOM that models impedance behavior in the frequency domain.

The third modeling approach is the fractional-order model (FOM) developed to enhance models’ precision [81]. FOMs are non-integer order differential equations that can better extract the parameters compared with the integer-order ECMs. Figure 9 illustrates FOMs reported in the literature. Figure 9a demonstrates a basic FOM including a series and a parallel resistance, a constant phase element, and a Warburg-lie element [38]. Figure 9b depicts an ECM consisting of three components in series [82]. The impedance behavior of the LiC is complex due to some kinetic steps. Thus, Z_P_ was employed in this model. The diffusion process and the porous nature of electrodes are the main factors for this transfer impedance. Figure 9c shows the same model with four parallel impedance branches (Z_P_) [83]. Figure 9d exhibits a developed FOM, in which the impedance is modeled by considering the ambient temperature at various currents [84]. Figure 9e shows an enhanced FOM regarding the influence of the cell’s temperature [85]. Figure 9f demonstrates an advanced FOM that models impedance behavior in the frequency domain that was validated for low current rates [86].

### 3.2. Lifetime Modeling

The lifetime of ESSs has an essential impact on EVs, as the total cost, safety, and reliability of each EV strictly depend on the lifetime of its ESS [87]. Thus, SoH estimation and prediction of the remained life of ESSs under real-time driving profiles represents an essential step to avoid issues [88]. Lifetime models have been developed in the literature to evaluate the long-term behavior of ESSs [89,90]. Therefore, developing a precise lifetime model is of high importance for ESSs that aim to operate under various working conditions for an expected duration. The lifetime model helps estimate the ESSs characteristics and determine the most severe working conditions during their predicted lifetime [91]. For this aim, developing a robust lifetime model requires a thorough understanding of aging processes and ESSs characteristics. Nevertheless, such comprehensive knowledge to predict the lifetime of ESSs is feasible for a limited number of applications [92]. Thus, understanding the main aging processes would be enough for lifetime modeling to predict the LiC’s end of life.

Almost all the LiC characteristics such as impedance parameters and capacity change over the lifetime of the cell. The main reason for this change is the aging of the cell under calendaring or cycling [31]. LiCs suffer from ESR rise and capacity fade when are exposed to calendaring and cycle aging processes. The main factors that influence the lifespan of LiCs include the type of anode material, the pre-lithiation level, inconsistency of the lithiated anode, and range of electrode potential [93]. The LiCs lifetime is reported as more than ten years [94]. However, the mentioned factors highly impact the lifetime of LiCs. The main factors that affect the degradation mechanism of LiCs are temperature, equivalent number of cycles (eq-cyc), depth of discharge (DOD), and current rate in charging and discharging scenarios. The main criteria to indicate the end of life degradation of LiCs are capacity reduction for 20% and ESR increase for 100% [95]. El Ghossein et al. [96] evaluated the aging mechanism of LiC for 20 months at high temperatures and various voltage levels, but the lifetime model was not developed. A lifetime model based on accelerated aging was developed that was capable of estimating the end of life. Nevertheless, the developed lifetime model was not comprehensive as the impact of the current rate was not applied in the modeling phases. Omar et al. [97] evaluated the impact of ESR growth and capacity reduction and concluded that ESR has more influence than capacity.

As is seen, the number of research articles is limited in LiCs degradation mechanisms and lifetime modeling, so an enormous potential can be seen here to fill this gap. The effect of temperature on the LiC cell’s components, including electrode materials and the electrolyte, should be investigated to thoroughly understand the impact of the operational temperature on the LiC cell’s performance. The target LiC cell in this dissertation has Lithium-Titanate-Oxide (LTO) as anode material. LTO is commonly used as the negative electrode, which features good capacity [26]. LTO, as the LiB anode, has high interfacial side reactivity with the electrolyte, which can lead to gas generation and poor cycling stability [98].

Huang et al. [99] investigated the temperature dependence of LTO degradation in LiBs. They have shown that the cell’s capacity retention would decrease at elevated temperatures; LTO could deliver up to 87.1% capacity retention in low C-rates (1C, 60 °C, 500 Cycles). However, with raising the C-rate, the capacity retention had drastically dropped to 20.9% (5C, 60 °C, 500 Cycles). Considering that high C-rates are in the interest of LiC applications, it is expected that controlling the temperature and keeping that in lower values could cause the LiC lifetime’s amelioration. Within another investigation, Yang et al. [100] evaluated the rate capability of LTO in a LiB cell at different temperatures from 0.5 °C to 20 °C and depicted 35 °C as the most efficient temperature for different C-rate values.

The electrolyte is also a vulnerable component against heat. Many common LiC electrolytes are based on carbonate solvents. Handel et al. [101] investigated the effect of high temperature on the degradation of pure Lithium-Hexafluoro-Phosphate (LiPF_6_)/carbonate-based electrolyte stored for 28 days at 60 °C and demonstrated that the electrolyte’s total impurity content had increased by about 2.5 times compared with the fresh electrolyte. Smart et al. [102] also reported degradation of LiPF6/carbonate-based electrolytes at elevated temperatures. To understand the aging factors, El Ghossein et al. [103] performed calendar accelerated aging tests on LiC cells cycled at 70 °C followed by a post-mortem study and concluded that pore-blocking at the positive electrode could decrease the capacitance values of the LiC cell and the main aging mechanism of the positive electrode is reported as the pore blocking of the activated carbon due to parasitic reactions between the functional groups present on its surface and the components of the electrolyte. According to what was mentioned, it could be concluded that internal heat, which would raise the operating temperature, is the most crucial factor in the LiC’s lifespan. Therefore, the application of LiCs requires a proper robust cooling system to extend the cell’s lifetime.

### 3.3. Thermal Modeling

All ESSs, including LiBs, EDLCs, and LiCs, produce heat during their operation [104,105]. Temperature is one of the most vital parameters that affect the performance of LiCs [106] by causing irreversible effects, including accelerated aging [107], impurity production [108], and solvent evaporation [109,110,111]. LiCs generate more heat with smaller ESR since they have been used in high power applications under high current rates [112]. Therefore, the lifetime and performance of LiCs significantly depend on their working temperature. In this context, an accurate thermal model is quite essential to design an optimized system to control the thermal behavior of LiCs [113]. An interdisciplinary knowledge of both thermal and electrical fields is required to develop an optimized LiC management system [114,115]. This work aims to develop a holistic 1D electro-thermal model for LiC technology coupled to 3D computational fluid dynamics (CFD) thermal model for further investigation of the temperature behavior of LiCs.

#### 3.3.1. Heat Transfer Modeling of LiCs

Before explaining the heat generation mechanism of LiCs, a thorough literature study on the LiC thermal model development is of high importance. Heat transfer modeling aims to determine the temperature evolution across the spatially discretized LiC, for which the thermal and geometric characteristics were identified [116]. Various heat generation types can be assumed, including conduction, convection, and radiation [117]. The heat conduction equation specifies the conduction, which is employed to model the temperature of the LiC’s surface and inside its layers [118]. In contrast, the dissipated heat from the LiC surface to the environment is due to convection heat transfer [119], and finally, the Stefan–Boltzmann law expresses the heat radiation [120].

This section deals with the heat transfer models developed for LiCs at the cell level or module level. The general equation for the heat dissipation of batteries was explained by Bernardi in 1985 [121]:

(4) $$P_{loss}=I\;\left(OCV-V_{t}\right)-I\;T\;\frac{\partial U}{\partial T}$$

where *I* [A], *OCV* [V], $V_{t}$ [V], and $\frac{\partial U}{\partial T}$ [V/K] represent current, open-circuit voltage (OCV), the terminal voltage of the cell, and the entropy coefficient (U denotes the cell’s voltage). The 1D thermal model for a cell stack was introduced by Pals and Newman in 1995 [122]:

(5) $$\rho C_{p}\frac{dT}{dt}=\left[\lambda_{x}\frac{\partial^{2}T}{\partial x^{2}}+\lambda_{y}\frac{\partial^{2}T}{\partial y^{2}}+\lambda_{z}\frac{\partial^{2}T}{\partial z^{2}}\right]+P_{loss}$$

where ρ [kg/m^3^] denotes the density, $C_{p}$ [J/kg·K] is the heat capacity, *T* [K] is the temperature, *λ* [W/m·K] represents thermal conductivity, and $P_{loss}$ [W/m^3^] represents the volumetric heat generation. Then, the heat flux from the cell to the ambient can be expressed as:

(6) $${\left.-\left[\lambda_{x}\frac{\partial^{2}T}{\partial x^{2}}+\lambda_{y}\frac{\partial^{2}T}{\partial y^{2}}+\lambda_{z}\frac{\partial^{2}T}{\partial z^{2}}\right]\right|}_{boundaries}={\left.h\left(T-T_{amb}\right)\right|}_{boundaries}$$

where h [W/m^2^.K], T [K], and$\;T_{amb}$ [K] represent the heat transfer coefficient, cell temperature, and ambient temperature. All the mentioned thermal parameters can be estimated using various methods, including the empirical approach, the electrical model for thermal behavior, and calorimetric evaluation. The first two methods have been used for LiCs, but the last one has never been employed for LiCs. For LiC cells, Equation (7) was updated to extract the thermal parameters of a 3300 F LiC [112]. Nevertheless, the heat source’s entropy change was not included. The same method was employed for a 1500 F LiC to predict the temperature of LiCs [58]. However, the model was unable to mimic the temperature of the cell for charging and discharging.

**Figure 10:**
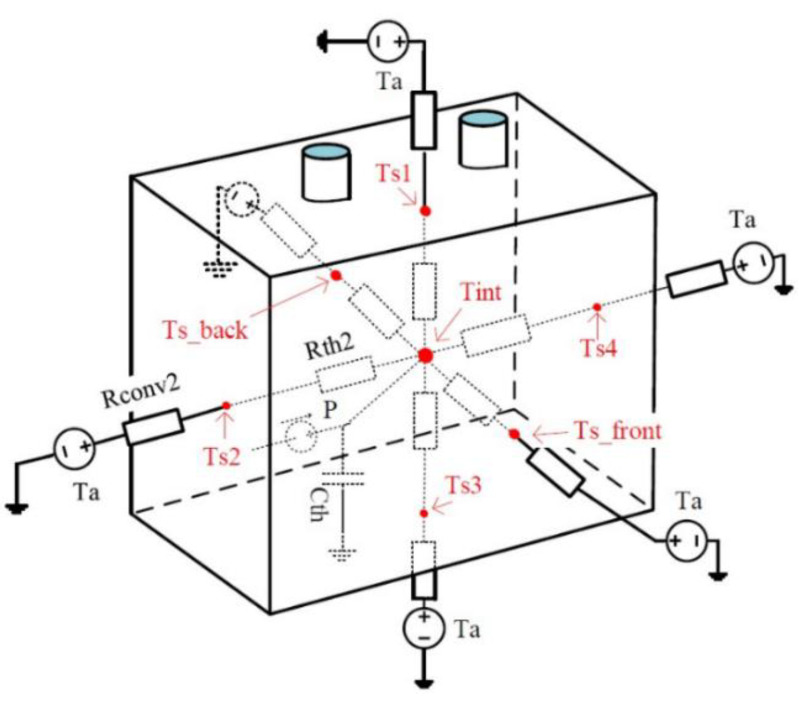
The RC branch used to predict the thermal behavior of LiC 3300 F cell that consists of four lateral sides (S_1_, S_2_, S_3_, and S_4_) front and back sides [59,125].

An RC branch was used for the first time in LiCs [59] for thermal parameters estimation using the least square fitting method. Reversible and irreversible heat sources were the main factor for temperature rise in this empirical approach. Nonetheless, the load profile was not high, showing the applicability of the model for high-power applications. Figure 10 depicts the RC branch used to predict the thermal behavior of LiC 3300 F cell. In the presented first-order thermal model, six branches of lumped thermal parameters are observed. T_s1_, T_s2_, T_s3_, and T_s4_ are the surface temperature for the lateral sides. T_s_back_ and T_s_front_ are the surface temperature of the back and front sides. T_int_ and T_a_ are the internal and ambient temperatures. R_th(i)_ and R_conv(i)_ are the thermal resistance from the middle point to side i, and convectional thermal resistance from side i to the ambient. The same model has been used in another work for thermal conductivity calculations in various directions for a prismatic 2300 F cell [123]. The extracted parameters were then utilized in another work for temperature uniformity analysis of LiCs [6].

It can be observed that a few models have developed a 1D thermal model for LiCs. However, their 1D model has not been coupled to a 3D thermal model to evaluate the accuracy of the extracted parameters. Only one study has developed a 1D model coupled to a 3D thermal model to investigate the thermal performance of a module of 12 LiCs under a forced-air cooling system [124]. However, the thermal performance of the proposed TMS was investigated under a 100 A current rate that is not considered a high-power application for LiCs, as the temperature of the 2300 F prismatic LiC that was investigated reaches around 40 °C after an hour of continuous operation. Therefore, a high current rate should be applied to such a cell to obtain the highest peak power deliverable by the 2300 F LiC cell.

The LiC structure is made for heavy-duty applications, where high power is demanded. Therefore, TMS development and modeling are indispensable to ensure the safe and reliable operation of such technology. Another critical challenge concerning LiCs would be the temperature non-uniformity, which is the main reason for electrical imbalance and SoC mismatch among the module cells [39]. Therefore, special attention should be given in designing a robust 3D thermal model to manage the temperature rise of LiCs during high charging and discharging. Table 1 lists all the developed 3D models for LiC cells. As can be observed, there is a massive potential in 3D thermal modeling for LiCs in high-power applications. As is seen in the literature study of LiCs, the 3D thermal behavior of the LiC cell is not analyzed and optimized comprehensively.

#### 3.3.2. Heat Generation Mechanism of LiCs

A summary of heat generation modeling methods for LiCs is listed in Table 2. By assuming the heat generation as non-uniform, the heat generation process can ideally be described by PDEs [138]. Nevertheless, solving such equations would be computationally inefficient since obtaining the microscopic parameters requires structural and material knowledge. On the other hand, by assuming the heat generation as uniform, the heat generation calculation is more straightforward since acquiring the equivalent macroscopic parameters is easier than the non-uniform heat generation [139]. Thus, uniform heat generation assumption is more suitable to investigate the EESs, since the heat flux per area has not been changed and our method is constant heat flux.

During the operation of LiCs, the temperature varies due to ion migration. This ion activity inside LiCs should be defined to measure the quantity and distribution of the heat generation. All the influenced factors in the heat generation of the LiC cell should be considered to develop a precise model to be able to estimate the electrical and thermal parameters of the cell. Moreover, the model should be able to estimate the end of life of the LiC since its lifetime is long and hard to reach within the experimental tests.

Based on the Bernardi equation [121], the generated heat of the cell incorporates the Joule loss, side reaction loss, and electrochemical reaction loss. The side reaction loss is neglected when the cell operates at reasonable temperature conditions [141]. To model the heat generation of the LiC, it should be considered that the cell’s generated heat consists of irreversible heat and reversible heat. The first part arises from the internal resistances of the electrolyte, electrodes, and collectors. In contrast, the second part stems from the entropy change of ions, plus Faradaic and non-Faradaic reactions [142]. In LiCs, the proportion of the reversible heat source is more extensive than the irreversible heat source due to Faradaic reactions, thus it cannot be neglected. In contrast, the proportion of reversible heat in EDLCs is significantly smaller than irreversible heat sources so that it can be neglected in the heat generation equations [125]. This would be the main difference between EDLCs and LiCs in terms of heat generation modeling. Thus, the Bernardi equation for LiCs can be written as:

(7) $$P_{loss}={\dot{q}}_{irr}+{\dot{q}}_{rev}=I\;\left(OCV-V_{t}\right)-I\;T\;\frac{\partial U}{\partial T}$$

As the temperature distribution of LiCs is non-uniform, the LiC’s heat sources are used separately for the tab domain and the cell domain. Equation (8) explains the heat source equation for the cell domain. For the tab domain, the heat generation can be explained as follows:

(8) $$P_{loss}=\frac{R\;I^{2}}{V_{tab}}$$

(9) $$R=\rho^{\prime }\;\frac{l}{S}$$

where *R* [Ω], *I* [A], *V_tab_* [V], $\rho^{\prime }$ [Ωm], *l* [m], and *S* [m^2^] denote the tab resistance, current, volume, resistivity, length, and cross-sectional area.

## 4. Application of LiCs

**Figure 11:**
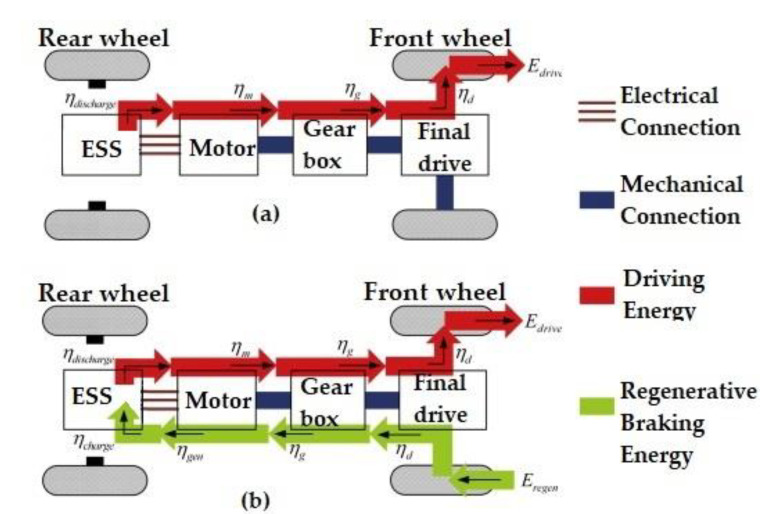
The Energy flow with and without RBS [151].

Currently, LiCs are employed for different applications such as electric vehicles (EVs) [143], flywheels for pulsed power applications [144], lightweight transportation systems [145], wind power plants [146], photovoltaic (PV) panels [147], power electronics [82], grid applications [55], railways [148], spacecraft and satellites [149], and power conditioning units (PCU) [150]. One of the most promising technologies counted as the end-user of LiC technology is the regenerative braking system (RBS), which collects energy from the braking of EVs or HEVs, trains, trams, and other types of automotive vehicles, as shown in Figure 11. The RBS bears low capabilities to restore the braking energy due to the limits of LiBs and EDLCs. However, LiCs can help this system regenerate and store higher amounts of energy for delivery to the vehicle’s traction based on the driver’s demand.

## 5. Conclusions

In this review paper, the working principle of LiBs, EDLCs, and LiCs was presented, focusing on the storage mechanism and chemical structure of LiCs. Then, the literature review was presented regarding the electrical, thermal, and lifetime models developed for LiCs. Various modeling methodologies, including the electrochemical modeling method, equivalent circuit models (ECM), and fractional order models (FOM), were listed for electrical model development. Aging factors and degradation mechanisms of LiCs were reviewed for the lifetime model development. Thermal model development, including 1D/3D thermal models, were reviewed, and heat transfer modeling and heat generation mechanisms of LiCs were presented. In the end, the application of LiCs was introduced.

## Figures and Tables

**Table 1:** The developed 3D models for LiCs in the literature with their type of TMS.

Ref.	Application	Cell/Module	Active	Passive	Hybrid
[123]	Thermal behavior analysis	Cell	-	✓	-
[126]	Cooling system design	Cell	-	✓	-
[124]	Cooling system design	Module	✓	-	-
[127]	Cooling system design	Cell & Module	-	✓	-
[128]	Cooling system design	Cell	✓	-	-
[129]	Uniformity analysis	Cell	-	✓	-
[130]	Cooling system design	Cell	✓	-	-
[131]	Cooling system design, uniformity analysis	Cell	✓	✓	✓
[42]	Lifetime analysis	Cell	✓	✓	-
[132]	Cooling system design, uniformity analysis	Cell	-	✓	✓
[133]	Holistic 1D/3D model, cooling system design	Cell	-	✓	✓
[134]	Cooling system design, uniformity analysis	Cell & Module	✓	-	-
[135]	Cooling system design, uniformity analysis	Cell & Module	✓	-	-
[136]	Cooling system design, uniformity analysis	Cell & Module	✓	✓	✓
[137]	Cooling system design	Cell	✓	-	-

**Table 2:** The heat generation estimation methods for LiCs in the literature.

Ref.	Heat Generation Mechanism	Heat Generation Assumption	Advantage	Disadvantage
[34]	Irreversible heat	Uniform	Low computational effort, easy parameters extraction	Low Precision
[59]	Irreversible heat	Uniform	Low computational effort, easy parameters extraction	Low Precision
[121]	Irreversible heat	Uniform	Low computational effort, easy parameters extraction	Low Precision
[140]	Reversible heat	Non-uniform	High precision, heat distribution description good	Heavy computational effort, harder parameter extraction
[121]	Reversible heat	Uniform	Low computational effort, easy parameters extraction	Low Precision
[140]	Reversible heat	Non-uniform	High precision, heat distribution description good	Heavy computational effort, harder parameter extraction

## Data Availability

Not applicable.
